# Tailoring Therapy: Hydrogels as Tunable Platforms for Regenerative Medicine and Cancer Intervention

**DOI:** 10.3390/gels11090679

**Published:** 2025-08-24

**Authors:** Camelia Munteanu, Eftimia Prifti, Adrian Surd, Sorin Marian Mârza

**Affiliations:** 1 Biology Section, Faculty of Agriculture, University of Agricultural Sciences and Veterinary Medicine Cluj-Napoca, 3-5 Manastur Street, 400372 Cluj-Napoca, Romania; eftimia.prifti@student.usamvcluj.ro; 2 Pediatric Surgery and Orthopedics, “Iuliu Hațieganu” University of Medicine and Pharmacy Cluj-Napoca, 400347 Cluj-Napoca, Romania; adisurd@elearn.umfcluj.ro; 3 Clinical Sciences Department, Faculty of Veterinary Medicine, University of Agricultural Sciences and Veterinary Medicine Cluj-Napoca, 3-5 Manastur Street, 400372 Cluj-Napoca, Romania; sorin.marza@usamvcluj.ro

**Keywords:** hydrogel, cancer, biomaterials, bio-functional

## Abstract

Hydrogels are water-rich polymeric networks mimicking the body’s extracellular matrix, making them highly biocompatible and ideal for precision medicine. Their “tunable” and “smart” properties enable the precise adjustment of mechanical, chemical, and physical characteristics, allowing responses to specific stimuli such as pH or temperature. These versatile materials offer significant advantages over traditional drug delivery by facilitating targeted, localized, and on-demand therapies. Applications range from diagnostics and wound healing to tissue engineering and, notably, cancer therapy, where they deliver anti-cancer agents directly to tumors, minimizing systemic toxicity. Hydrogels’ design involves careful material selection and crosslinking techniques, which dictate properties like swelling, degradation, and porosity—all crucial for their effectiveness. The development of self-healing, tough, and bio-functional hydrogels represents a significant step forward, promising advanced biomaterials that can actively sense, react to, and engage in complex biological processes for a tailored therapeutic approach. Beyond their mechanical resilience and adaptability, these hydrogels open avenues for next-generation therapies, such as dynamic wound dressings that adapt to healing stages, injectable scaffolds that remodel with growing tissue, or smart drug delivery systems that respond to real-time biochemical cues.

## 1. Introduction

### 1.1. Hydrogels: Enabling Precision Medicine

One well-established and quickly developing area of medicine is precision medicine, which customizes treatments according to a patient’s genetic, environmental, and lifestyle factors. These findings suggest that hydrogels’ special qualities make them a potential platform for this topic [1]. Adding to their natural biocompatibility—which, because of their structural resemblance to the extracellular matrix of the body, gives them a promising platform for precision medicine—hydrogels’ real worth is found in their capacity to be created as active instead of passive materials. The extracellular matrix (ECM) of the body’s tissues is quite similar to the porous, water-rich structure of hydrogels. In order to integrate with the body without triggering an immunological reaction, this biocompatibility is essential [2].

Since their special qualities may be designed to deliver therapeutic substances precisely where they’re needed without harming healthy tissues, hydrogels are a perfect platform for precision medicine. Highly specialized delivery methods are needed for precision medicine, which customizes therapies to meet the needs of each patient [3]. Hydrogels, which are networks of three-dimensional polymers that can hold much water, can be made into “smart” materials that react to specific signals in the body, like temperature changes, pH changes, or the presence of certain enzymes or biomarkers, which are frequently signs of inflammation or cancer [4]. Drugs or other medicines can be encapsulated within these stimuli-responsive hydrogels to release exclusively at the intended place, reducing systemic side effects and guaranteeing a localized, long-lasting, and highly effective therapeutic effect [5].

The main contention is that hydrogels are not passive materials. It is possible to design them to be “tunable” and “smart”, which allows for precise adjustment of their characteristics [6]. This includes both mechanical and chemical properties, which allow them to be functionalized with particular biological molecules, such as growth factors or peptides, to control cell behavior, and to match the stiffness of a target tissue (for example, soft for brain tissue or firm for cartilage) [7].

The section contends that by facilitating targeted, localized, and on-demand therapy, hydrogels provide a substantial benefit over conventional systemic drug delivery, which impacts the entire body. A distinguishing feature of precision medicine is its specificity [8]. The production of hydrogels with tailored characteristics is essential for expanding their use in biomedical applications, including diagnostics (bio detection and bioimaging), therapeutic delivery (drugs, immune therapeutics, and vaccines), wound dressing and skin materials, cardiac complications, contact lenses, tissue engineering, and cell culture [9,10].

Hydrogels play an important role in cancer therapy because they allow for the targeted and prolonged release of chemotherapeutic medicines, immune therapeutics, and other anti-cancer agents directly at the tumor site. This focused strategy considerably enhances therapeutic effectiveness while minimizing the severe systemic toxicities that are often associated with traditional therapies [11].

Furthermore, “smart” hydrogels may be made to react to certain internal (e.g., pH or temperature) or exterior (e.g., light) stimuli found in the tumor microenvironment, enabling on-demand and remotely controlled drug release [12]. They also contribute to the in vitro modeling of the tumor microenvironment, which helps elucidate cancer cell behavior and create novel therapeutics [13]. Typically, hydrogels are created by crosslinking polymeric chains or other macromolecular components, such peptides or colloidal assemblies, to create a stable 3D network that can hold a great deal of water. [14]. This may be accomplished by physical crosslinking (ionic contacts, hydrogen bonding, etc.) or chemical crosslinking (free radical polymerization, click chemistry, condensation processes, etc.). The approach employed is determined by the required attributes, which include mechanical strength, degradation rate, and responsiveness. Dual-sensitive hydrogels, for example, may be engineered to react to changes in temperature and pH, allowing for precise drug release control [15].

According to the medical uses, they may be developed as injectable hydrogels that are injected in liquid form and subsequently gel in situ inside the body, which is very beneficial for minimally invasive delivery to specific areas such as tumors. They may also be used as “bio-inks” in 3D bioprinting to create complex tissue models or scaffolds with precise topologies for tissue engineering purposes. Hydrogels are used topically for external usage, such as wound treatment, in the form of sheets, gels, or sprays [16].

Hydrogels are significant because they are biomimetic, meaning they closely resemble real tissues, ensuring great biocompatibility [17]. Their adjustable features enable the adjustment of mechanical strength, degradation rate, and swelling behavior to meet a wide range of medical applications [18]. The capacity to create regulated and stimuli-responsive release of therapeutic drugs is crucial for precision medicine since it improves effectiveness while lowering adverse effects. This plasticity in structure and function, together with the possibility of minimally invasive distribution, establishes hydrogels as critical materials with enormous promise for the future of medicine and cancer therapy [19].

### 1.2. The Aim of the Review

This review provides a comprehensive and critical examination of recent advancements in hydrogel-based systems for regenerative medicine and cancer therapy. It offers an integrated perspective that connects material design with therapeutic application, placing particular emphasis on the development of tailored hydrogel platforms. The discussion focuses on how precise modulation of hydrogel properties such as mechanical strength, degradation kinetics, bio-adhesiveness, and responsiveness to external stimuli can address current biomedical challenges and enhance clinical efficacy. By consolidating emerging strategies in both engineering and application, this review aims to advance the understanding of hydrogel functionality in complex therapeutic contexts.

This review emphasizes recent strategies in hydrogel engineering that focus on the precise modulation of key material properties to meet the complex requirements of regenerative medicine and cancer therapy. Beyond commonly addressed features such as mechanical strength, degradation rate, bio-adhesion, and stimuli responsiveness, special attention is given to often overlooked but critical factors like scalability and biocompatibility. These characteristics play a vital role in ensuring optimal performance and clinical readiness. A lack of tunability in these areas continues to hinder the translation of hydrogels from laboratory research to practical therapeutic use, particularly in drug delivery and tissue regeneration.

## 2. Tunable Hydrogel Fundamentals

### 2.1. Material Selection and Crosslinking

**Figure 1:**
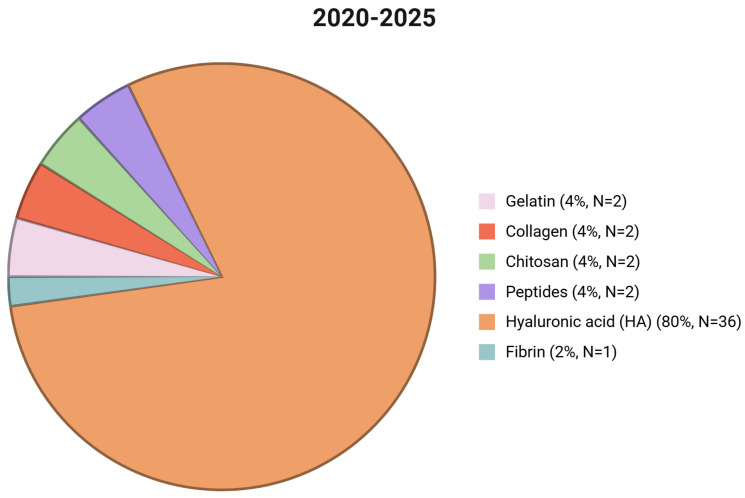
Distribution of commonly used natural polymer hydrogelators reported in the reviewed literature from 2020–2025 (*n* = 106 articles; *N* = the number of articles with specific hydrogels).

Hydrogels are typically described as three-dimensional, hydrophilic networks capable of absorbing and retaining substantial amounts of water or biological fluids while maintaining structural integrity due to physical or chemical crosslinking [20]. Their preparation entails the careful selection of building components, which may be sourced from either naturally occurring or synthesized hydrogelators. The hydrogel’s mechanical, biological, and physicochemical characteristics are heavily influenced by the component materials used [10,21]. Collagen, chitosan, peptides, hyaluronic acid (HA), alginate, fibrin, gelatin, elastin, and silk protein are some of the most commonly used natural polymers. A statistical analysis of the research reviewed from 2020–2025 (*n* = 106 papers) found that HA was the most frequently reported natural polymer hydrogelator, with 36 publications. Collagen, chitosan, peptides, and gelatin were all referenced in two studies, but fibrin was only mentioned once. The chosen recent literature did not include reports of alginate, elastin, or silk protein. These findings show a substantial research emphasis on HA-based hydrogels in recent years, reflecting their flexibility, biocompatibility, and proven importance in biomedical applications (Figure 1).

In addition to bulk material choice, the incorporation of specific functional groups or degradable linkers such as enzyme-sensitive sequences or hydrolysable bonds further tailors the hydrogel’s behavior for targeted biomedical applications. Polyacrylamide and polyvinyl alcohol (PVA) are well-known synthetic polymers [22]. These complex polymeric networks are formed via a variety of crosslinking processes, which may be generally divided into chemical and physical techniques. Chemical crosslinking essentially entails the establishment of strong covalent connections between polymer chains, a process that typically yields hydrogels with greater mechanical strength and longer-term stability compared to physically crosslinked systems [23]. Common chemical crosslinking methods include free radical polymerization, photo-crosslinking, click chemistry, and enzymatic crosslinking [24].

Citric acid (CA) serves as a chemical crosslinking agent for carboxymethyl cellulose (CMC) and polyvinyl alcohol (PVA) hydrogels, creating stable ester linkages that characterize the hybrid polymeric network [22]. Conversely, physical crosslinking depends on non-covalent interactions, including ionic interactions, hydrogen bonding, hydrophobic associations, van der Waals forces, and physical entanglement. These interactions are mostly reversible, a trait that often enhances the biocompatibility and biodegradability of hydrogels, facilitating their reversible construction under physiological settings [23]. Methylcellulose-based hydrogels remain liquid at ambient temperature, enabling plasma treatment, and then solidify at physiological temperature, while alginate-based hydrogels harden in aqueous environments, rendering them very suitable for injectable applications [25].

Crosslinking is essential for determining the overall characteristics and varied uses of hydrogels. Its principal function is to provide dimensional stability and inhibit hydrophilic polymer chains from disintegrating in watery settings. The crosslinking density is a vital quantity that directly affects many essential hydrogel features, such as swelling behavior, mechanical strength, and water retention capacity [26]. An increase in crosslinking density often results in a reduction in equilibrium swelling and stretchability while concurrently enhancing mechanical strength and stiffness. Moreover, crosslinking is crucial for providing enough mechanical strength to affect drug transport and for accurately customizing the release characteristics of encapsulated bioactive agents, thereby facilitating regulated drug administration [27]. The selection of crosslinking technique and density is a crucial design trade-off that directly balances the immediate need for structural integrity with the long-term biological demands for suitable degradation kinetics. Chemical crosslinking ensures great stability and strength, while physical crosslinking gives reversibility and often enhanced biocompatibility and biodegradability [10,23]. In tissue engineering applications, it is essential that the hydrogel’s breakdown rate aligns closely with the pace of new tissue formation [24]. In drug delivery, the release profile is meticulously regulated by the hydrogel’s swelling and breakdown properties [28]. Excessive stability from robust chemical crosslinks may obstruct normal tissue remodeling, restrict cell migration, or inhibit the full release of therapeutic medicines, potentially resulting in persistent inflammation or material buildup in vivo. Conversely, inadequate crosslinking may jeopardize the scaffold’s functional integrity or lead to premature drug release. The discipline is advancing towards more intelligent material design, shown by complex tuning mechanisms like self-cleaving linkers that accurately synchronize drug release with hydrogel degradation, offering an advanced method to dynamically adjust to the biological environment [29].

Excessive chemical crosslinking in hydrogels can create overly stable matrices that hinder normal tissue remodeling, restrict cell migration, or prevent the complete release of therapeutic agents, potentially leading to chronic inflammation or residual material accumulation in vivo [30]. To address these limitations and meet the demands of multi-phase therapeutic strategies, recent research has developed hierarchical hydrogel architectures capable of precise spatial and temporal regulation of bioactive compound release. These systems often embed nanoscale or microscale gel particles within a larger bulk hydrogel matrix, allowing drugs to be compartmentalized according to their release kinetics—such as targeting distinct phases of cancer therapy or wound healing—while enabling independent control of diffusion and degradation properties [31].

Several design approaches illustrate this concept. Embedding PLGA-based microspheres into injectable hydrogels enables sequential release of multiple drugs over programmable time frames [31]. Nanoparticle–hydrogel composites (NP-gels) integrate nanoparticles within the hydrogel network, providing fine-tuned release kinetics via multi-scale structuring [32]. In another example, ordered micro–nano architectures have been used in liver cancer-on-a-chip models to create structured hydrogels for precision drug testing [33]. More recently, HA-modified ZIF-8 nanoparticle systems have demonstrated sustained, multi-level release of agents such as lidocaine, further highlighting the potential of hierarchical designs for extended and controlled delivery [34]. These strategies are particularly advantageous for complex therapeutic scenarios, such as delivering an initial antibiotic burst from surface particles followed by sustained anti-inflammatory release from embedded microspheres, offering a powerful platform for advanced, multi-phase treatment regimens.

### 2.2. Physico-Chemical and Mechanical Control

The mechanical characteristics of hydrogels are well studied and are crucial for their effective use in several biomedical domains, such as ligament and tendon healing, wound dressing, and tissue engineering [35]. The mechanical characteristics of the hydrogel are also greatly influenced by the degree of porosity; in general, scaffold stiffness decreases as porosity rises. For example, 5 µm is suitable for neovascularization, but 100–350 µm is favored for bone regeneration [36]. Attributes including fracture strength, elastic modulus, toughness, and nonlinear elastic behaviors such as strain softening or strain stiffening may be markedly improved and precisely tuned through advanced methodologies [36]. These nonlinear attributes are critical in many biomedical contexts, for example, in load-bearing cartilage scaffolds where strain stiffening prevents over-compression or in vascular grafts where strain softening facilitates compliance under pulsatile flow [37]. A direct but effective method entails nonsolvent-quenching combined with thermal annealing, resulting in hydrogels with hierarchical architectures characterized by fibrous networks and exceptional mechanical properties [38].

The mechanical properties of hydrogels arise from their network morphology and can generally be categorized into two primary types:

1. Rubber-like hydrogels—often modeled by classical rubber elasticity and viscoelasticity theories; they are characterized by significant extensibility under low stress and full recovery driven primarily by entropic elasticity [39]. Examples include polyacrylamide, PEG (polyethylene glycol)-based, and PDMS (polydimethylsiloxane)-based hydrogels;
2. Fibrillar hydrogels—such as collagen, fibrin, silk fibroin, or synthetic fiber-forming systems, where the mechanical response is dominated by enthalpic elasticity. In these materials, deformation resistance is due to stretching of semi-flexible fibers rather than entropy-driven chain retraction [40,41]. Fibrillar networks often exhibit strain stiffening, a nonlinear behavior crucial in mimicking extracellular matrix mechanics [36,42].

Across both morphologies, several key determinants influence mechanical performance and tunability:

- Crosslinking density—higher density increases rigidity but may reduce extensibility [43];
- Monomer molecular weight—larger monomers or longer polymer chains yield stronger, more resilient networks [44];
- Monomer concentration—higher concentrations create denser networks, enhancing strength and viscoelasticity [45];
- Monomer composition—specific chemistries (e.g., hydrophobic domains or ionic groups) can improve toughness, energy dissipation, or responsiveness [46].

The mechanical properties of hydrogels are elucidated using the principles of rubber elasticity and viscoelasticity. In their expanded form, most hydrogels have rubber-like properties, marked by significant extensibility under little stress and full recovery facilitated by entropic variations in addition to time-dependent viscoelastic responses [23]. Crucial determinants that significantly affect and facilitate the modulation of mechanical properties encompass crosslinking density (with elevated density typically augmenting rigidity), monomer molecular weight (greater molecular weight fosters stronger, more resilient networks), monomer concentration (increased concentrations yield denser networks, enhancing strength and viscoelasticity), and monomer composition (particular monomers can improve properties via hydrophobic interactions or ionic bonding) [22].

The rate and degree of hydrogel swelling are essential factors that directly influence the release patterns of encapsulated solvents and drugs from polymeric networks. Swelling kinetics are significantly affected by various design parameters, such as the crosslinking ratio, the chemical composition of the polymer, the ionic environment, and the conditions of synthesis [47]. The equilibrium swelling, dimensional changes, and drug release patterns of hydrogels are influenced by the balance of hydrophilic and hydrophobic components, the degree of crosslinking, the degree of ionization, and interactions with counterions [19]. Mathematical models, including Fick’s law with position-dependent diffusion coefficients and theories of concentration-dependent diffusion, are utilized to predict and regulate drug release from swelling-controlled networks [48].

One essential and much-desired improvement for hydrogels, especially in biomedical applications, is controlled deterioration. In tissue engineering applications, the degradation rate of the hydrogel must align with the rate of new tissue growth, thereby providing ongoing support while facilitating natural tissue remodeling. Hydrogels can be designed to degrade via multiple mechanisms, such as hydrolysis, enzyme-mediated processes, or a combination thereof [49,50,51].

The degradation rate can be accurately regulated by altering the chemistry of the degradable linker, such as the faster degradation of ester linkages from lactic acid compared to caprolactone, or by integrating enzymatically susceptible linkages with designated amino acid sequences. More highly crosslinked gels typically demonstrate extended degradation times [52]. Advanced methods utilize self-cleaving β-eliminative linkers to independently adjust drug release and hydrogel erosion rates across a broad spectrum, ensuring optimal drug release prior to the complete erosion of the gel. Porosity is a crucial structural component that significantly affects how well hydrogels work because it controls their transport characteristics, which are necessary for uniform cell distribution, effective diffusion of nutrients and oxygen, and local angiogenesis after implantation [53].

Different pore sizes are best suited for the regeneration of different tissues. Solvent casting/particle leaching, gas foaming, electrospinning, freeze drying (lyophilization), and sophisticated microfabrication methods including soft lithography and fast prototyping are methods for carefully regulating hydrogel porosity and microarchitecture. In order to guide tissue development and encourage vascularization, these techniques allow for the customization of pore size, total porosity, and complex microarchitectural characteristics like interconnecting microchannels [53]. The many excerpts explain how a variety of factors may be used to independently adjust swelling, porosity, and mechanical qualities. It is generally known that pore size and scaffold stiffness have a significant impact on cell behavior, including adhesion, proliferation, and differentiation [54].

Similarly, the swelling behavior and porous structure of the hydrogel directly regulate the kinetics of drug diffusion and release [55]. This suggests that rather than being separate factors, these characteristics are intricately linked and together determine the dynamic milieu for encapsulated cells as well as the release profile of therapeutic medicines. For example, increasing porosity to improve nutrition flow may unintentionally reduce the stiffness of the scaffold, which may change the pathways involved in cell differentiation [23]. On the other hand, even while a stiff, highly crosslinked hydrogel offers superior mechanical support, it may hinder swelling and therefore drug diffusion. This draws attention to a multifaceted, intricate design space where improving one characteristic may call for compromises or the integration of design elements with others [47]. Therefore, a comprehensive and integrated approach to hydrogel design is necessary to provide genuinely “tailored” treatment. To precisely direct cellular responses, encourage desirable tissue regeneration, and regulate drug kinetics in vivo, researchers must take into account the dynamic interaction of these mechanical and physicochemical features (Table 1). This calls for the creation and use of sophisticated mathematical models and advanced characterization techniques such as 3D pore reconstruction in order to precisely anticipate and regulate these intricately linked interactions [56].

### 2.3. Smart and Bio-Functional Hydrogels

**Figure 2:**
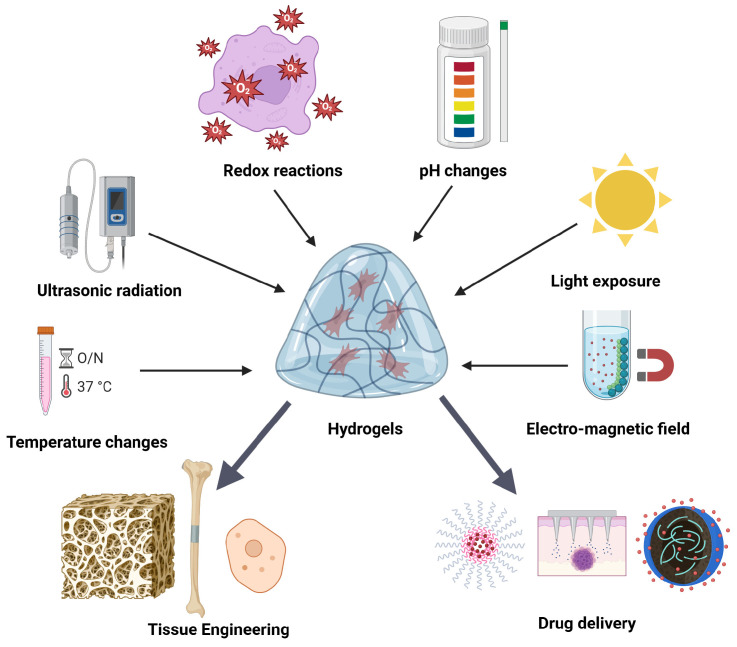
Representation of various external stimuli that affect bio-functional and smart hydrogels. This figure illustrates the range of physicochemical stimuli that can induce responsive and intelligent hydrogels to undergo functional changes. Redox reactions (represented by ROS), ultrasonic radiation, pH changes (indicated by the pH scale), temperature changes (in °C), light exposure, and the use of electric and magnetic fields are examples of these stimuli. The smart and bio-functional properties of these hydrogels in various applications, particularly in tissue engineering and controlled drug delivery, are primarily dependent on their ability to respond to specific cues.

Smart hydrogels, often known as stimuli-responsive hydrogels, are an advanced category of materials designed to undergo certain structural alterations, such as expansion or contraction, in direct response to diverse external stimuli. The stimuli may be physicochemical, including variations in pH, temperature, light, electric and magnetic fields, redox chemical processes, and ultrasonic irradiation [57] (Figure 2).

Alternatively, they may refer to particular biological things, like tiny molecules, proteins, enzymes, or metal ions. This intelligent responsiveness is an essential characteristic that allows for precisely regulated drug release and supports dynamic applications in intricate biological settings [23].

Various categories of smart and bio-functional hydrogels have been developed. Self-healing hydrogels possess the capability to autonomously repair structural damage, thereby prolonging their functional lifespan and improving reliability in dynamic biomedical applications, including wound healing and tissue scaffolding. Their notable self-repair capabilities are mainly due to the inclusion of dynamic covalent bonds (e.g., Schiff base linkages, disulfide bonds, etc.) or various non-covalent interactions (e.g., hydrogen bonding, ionic interactions, host–guest chemistry, etc.) in their polymeric networks [52].

Tough hydrogels mitigate the mechanical fragility typically found in conventional hydrogels and are designed with superior mechanical properties. Their robustness facilitates successful application in load-bearing contexts, including cartilage regeneration and artificial ligaments [58]. Common design strategies encompass the development of double-network (DN) structures, characterized by two interpenetrating polymer networks, and the deliberate integration of reinforcing agents such as nanoparticles or nanofibers [59].

Hydrogels function as practical three-dimensional structural templates, closely resembling the ECM and offering essential support for cell adhesion, migration, proliferation, and differentiation. To preserve cell viability and function, they make it easier for cells to encapsulate under gentle, cell-friendly gelation conditions. Bio-functional hydrogels can be designed to include specific bioactive cues, such as Arg-Gly-Asp (RGD) sequences to improve cell adhesion or matrix metalloproteinase (MMP)-sensitive linkers for facilitating cell-mediated degradation and tissue remodeling [60,61]. One of the most recent developments is the 3D printing of highly customizable hydrogel scaffolds, which enable intricate designs and sophisticated mechanical characteristics that may be tailored to each patient. Moreover, hydrogels based on gelatin have become a potential class of biomaterials for peripheral nerve regeneration because they may facilitate axonal elongation and allow for the targeted and prolonged release of stem cells or neurotrophic factors [62].

The development of smart and bio-functional hydrogels represents a huge step forward, allowing for dynamic response to diverse stimuli (e.g., pH, temperature, and light) as well as the inclusion of unique biological signals. These sophisticated hydrogels, including self-healing, resilient, and hybrid systems, provide unparalleled regulation of drug delivery, cell encapsulation, and tissue engineering applications. The synergistic integration of intelligent functionality with biomimicry facilitates the development of advanced biomaterials that can actively sense, react to, and engage in intricate biological processes [63].

### 2.4. Limitations of Conventional Hydrogels

Despite their versatility, conventional hydrogels face several intrinsic limitations that can hinder their clinical translation. One major drawback is their poor mechanical strength, which makes them prone to deformation or fracture under physiological stresses, limiting use in load-bearing tissues such as cartilage or tendons [47,64]. Many hydrogels also exhibit uncontrolled swelling, leading to rapid drug release and loss of dimensional stability [65]. Furthermore, their degradation rates are often difficult to match with tissue regeneration timelines, resulting in either premature loss of mechanical support or excessive persistence that impedes tissue remodeling [66]. Traditional hydrogels may also suffer from low bioactivity, as their inert polymer networks lack the biochemical cues needed to promote cell adhesion, proliferation, and differentiation [45]. In drug delivery, limitations include burst release profiles, poor protection of sensitive therapeutics, and minimal capacity for sequential or targeted release [67]. Addressing these challenges has driven the development of advanced hydrogel systems—including physically and chemically hybridized networks, hierarchical architectures, and stimuli-responsive formulations—that aim to overcome these constraints and enable precise, application-specific performance [53].

## 3. Tunable Hydrogels in Regenerative Medicine

Hydrogels are advanced scaffolds and delivery systems used in regenerative medicine that can be designed to replicate the natural cellular environment, thereby directing tissue growth and healing. Their adaptability enables the following:

1. Tailored mechanical properties: the mechanical properties of the body’s tissues vary greatly, ranging from tough bone to soft brain matter;
2. Controlled degradation rates: the hydrogel scaffold must break down at a rate that precisely corresponds to the rate at which new tissue is growing for successful tissue regeneration [8];
3. Accurate biochemical signaling: hydrogels can be designed to contain and release specific bioactive substances, including peptides, growth factors, or cytokines, in a controlled and prolonged manner;
4. Cell delivery and encapsulation: hydrogels offer a supportive and safe environment for the delivery and encapsulation of cells, including stem cells;
5. Architectural customization: hydrogel scaffolds with incredibly complex and accurate designs that replicate the intricate structures of natural tissues and organs can be created using advanced fabrication techniques, such as 3D bioprinting [68].

### 3.1. Tissue Engineering Scaffolds

**Figure 3:**
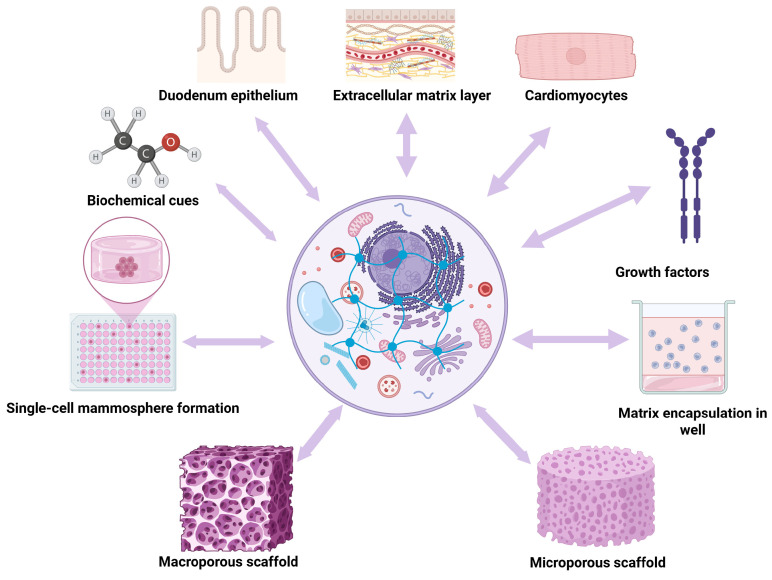
Schematic illustration of scaffolds for tissue regeneration using hydrogel-based tissue engineering. Hydrogels are flexible scaffolds that can be used to encapsulate a range of medicinal substances. For tissue engineering applications, they can encapsulate various stem cell types, such as adult stem cells, induced pluripotent stem cells (iPSCs), and human embryonic stem cells (hESCs). Hydrogels can also transport growth factors, transcription factors, and nanoparticles to support tissue repair and cellular differentiation. The regeneration of different tissues, including skin, muscle, and bone, is facilitated by these integrated approaches.

In regenerative engineering, creating a scaffold that can replicate natural ECM and guide stem cells to produce functional tissues is a significant problem. Hydrogels are among the most promising biomaterials in this regard because of their high water content, biocompatibility, and ease of tuning to replicate the characteristics of the extracellular matrix [69]. To repair various tissues through regenerative engineering, numerous types of hydrogels with enhanced chemical and physical properties have been recently developed [70] (Figure 3).

For instance, to systematically control cell behavior, including migration, proliferation, and differentiation, multiple biophysical cues, such as stiffness, porosity, and degradation, have been integrated into hydrogel scaffolds in a spatiotemporally controlled manner [71,72].

To enhance the biocompatibility and functionality of hydrogels, numerous sophisticated chemical techniques as well as the addition of functional ingredients have been suggested [73]. It is possible to successfully regenerate complex tissues by imitating the different native extracellular matrix compositions and architectures. By expanding our understanding of cell–matrix interactions at the nanoscale and facilitating the fabrication of increasingly complex multicellular designs, modern nano- and microtechnologies can significantly support regenerative engineering. Hydrogel scaffolds, for instance, have been utilized to study cell behavior by spatially patterning them with various nanoscale biophysical and pharmacological stimuli [74]. Recently, new polymer-processing technologies have been integrated to create an ECM–mimetic nanofibrous hydrogel with customizable architecture and mechanics [75].

In this industry, collagen-based hydrogels are frequently utilized due to their cell-binding and biocompatibility properties. The quality of the engineered skin is impacted by its drawbacks, which include immunogenicity, rapid deterioration, inadequate mechanical strength, and shrinkage during fibroblast culture [76,77]. Crosslinking techniques and composite systems have been developed to improve the mechanical characteristics and stability of collagen hydrogels to get around these problems [78,79,80,81].

Granular hydrogels (GHs), composed of packed hydrogel microparticles (HMPs), have garnered considerable interest recently in various domains, including bioprinting, drug delivery, and tissue engineering [82,83,84]. Along with injectability for minimally invasive applications and customizable mechanical characteristics through changes in the size, content, and crosslinking density of HMP, these hydrogels have high porosity and permeability, which encourage cell migration and ease the exchange of nutrients and waste. Additionally, studies have demonstrated that the immunological response to GHs in vivo differs from the reaction to traditional bulk hydrogels [85,86]. However, because of their weak interparticle pressures, classic GHs—which rely on physical jamming effects—frequently lack self-supporting structures, which restricts their direct application in tissue scaffolds. Two approaches—bioprinting in a gel bath and utilizing physical or chemical techniques to enhance HMP interactions—have been developed to overcome this constraint [87,88,89,90]. The fact that self-supporting behavior is not enough to ensure mechanical compatibility for in vivo applications is nevertheless recognized. The physiological conditions (e.g., tissue deformation, blood pressure, or muscular action) must be taken into account when determining the critical stress necessary to break or distort the gel under such situations. It is currently accepted that a more thorough comparison with the spectrum of stresses anticipated during in vivo application would provide greater context even if the gel’s yield stress and mechanical integrity have been described under laboratory circumstances [91]. It is well known that quantitative assessments of physiological stress differ based on the application site. For instance, arterial pressures might translate into dynamic strains of roughly 16 kPa (120 mmHg) or more, but soft tissues may experience stress in the range of 1–10 kPa [92].

Perhaps future research will try to compare the mechanical characteristics of the gel to particular physiological stress ranges that are pertinent to its intended application. It is reasonable to assume that including such estimations would improve the discussion and the applicability of the conclusions in real-world situations.

Therefore, we can conclude that hydrogels play a crucial role in regenerative medicine by providing highly adaptable scaffolds and delivery systems that replicate the cellular environment found in the body. Their ability to precisely regulate mechanical characteristics, rates of degradation, biochemical signaling, cell delivery, and architectural design is one of their main advantages. Even though ongoing developments, especially in the area of extracellular matrix mimicry, hold considerable promise, problems still exist with conventional materials (Table 2). Essentially, “conventional materials” refers to the typical norm that advanced hydrogels were created to surpass, providing answers to the significant constraints concerning tissue integration, flexibility, and dynamic biological signaling.

### 3.2. Controlled Release Systems/Polyethylene Glycol (PEG)

Fundamentally, tunable hydrogels are clever networks of polymers made to change predictably in response to particular stimuli. A variety of physiological stimuli, including variations in pH, temperature, or ionic strength, as well as the presence of specific enzymes or biomolecules at an injury or disease site can cause these stimuli-responsive hydrogels to transition from a collapsed, compact state to a swollen, expanded state. Their capacity for controlled release is based on this “smart” behavior [93]. These hydrogels efficiently protect encapsulated therapeutic agents from external degradation, such as harsh proteolytic enzymes, when they are collapsed. The hydrogel expands in response to the right stimuli, which enables the localized release of the encapsulated medications at the exact moment and location that they are required. By focusing the treatment at the target site, this on-demand release mechanism maximizes therapeutic efficacy, minimizes systemic exposure, and minimizes potential side effects [94].

Tunable hydrogels enable controlled release using a range of complex techniques, going beyond basic swelling and collapsing. The “pro-drug” technique is a sophisticated method that works exceptionally well with highly hydrophilic materials, such as polyethylene glycol (PEG) hydrogels [95]. The reason PEG was selected for discussion in this section is that it is a popular and perfect component for the creation of controlled drug delivery systems due to its many features, including biocompatibility, tunable degradation, and the capacity to be modified with different chemical functions. Using specialized pendant functional groups, therapeutic molecules are covalently tethered within the hydrogel network rather than just entrapped. Most importantly, the drug molecule and the hydrogel backbone are joined by biodegradable linkers [96]. These linkers are designed to decompose in a specific manner, either through enzymatic cleavage (cleavage by particular enzymes) or hydrolytic reaction (reaction with water). Scientists can precisely control the rate of drug liberation and release by manipulating the chemistry of these linkers, enabling sustained, long-term delivery profiles tailored to the unique requirements of tissue regeneration [97].

The structure–property relationships of PEG-based hydrogels have been made clear by numerous basic investigations, particularly those about equilibrium swelling, mechanics, and transport properties [20,98]. These investigations led to the development of a variety of PEG based hydrogel systems for the controlled delivery of a wide range of biomolecules, from large biomacromolecules like proteins, peptides, and nucleic acids to small molecular weight medications. Design criteria for controlled release in PEG based hydrogels can vary significantly from one application to another due to the wide range of delivered molecules’ sizes and chemistry. However, when creating PEG hydrogels for applications requiring controlled release, two key considerations are the therapeutics’ stability and accessibility. The ability of PEG hydrogels to deliver therapeutics at the appropriate dosage is necessary to achieve the intended therapeutic efficacy in vivo or in vitro.

The process of drug loading, the drug’s size and molecular makeup, and the dosage and release profile required for a given therapeutic application are some of the variables that affect the mechanisms by which drugs are released from PEG hydrogels. Generally speaking, diffusion-controlled, swelling-controlled, and chemically controlled delivery are the primary molecular release mechanisms found in PEG hydrogels. For in-depth mechanistic explanations of these release mechanisms, readers are directed to other reviews [20,99]. The high permeability of PEG hydrogels makes it challenging to control the release kinetics of small-molecular-weight therapeutics, such as synthetic drugs, small peptides, and proteins. Tuning the gel permeability by varying the network crosslinking density is a simple method of controlling molecular release kinetics. However, this method is ineffective for regulating the release of small molecules because it relies on size exclusion. For the majority of tissue engineering applications, it is also undesirable because a higher crosslinking density frequently indicates decreased hydrophilicity and, consequently, decreased cytocompatibility. Therefore, to preserve the preferred hydrophilic environments that PEG hydrogels offer, other controlling mechanisms must be strategically implemented [20,100,101,102]. These cleverly created stimuli-responsive hydrogels can change from a collapsed to a swollen state in response to physiological stimuli (such as pH, temperature, and ionic strength). The encapsulated medications are shielded from externally damaging elements, such as proteolytic degradation, when they are collapsed. When the correct stimuli is applied, the encapsulated medications are then released in their enlarged state.

The “pro-drug” technique is an additional method of drug delivery that preserves the preferential hydrophilicity of PEG hydrogels. This method utilizes pendant functional groups to covalently tether therapeutics within PEG hydrogels. Predetermined liberation and release rates are made possible by the addition of degradable linkers between the drug and the tether. Numerous linker degradation mechanisms, such as enzymatic and hydrolytic degradation, have been proposed [103,104].

These hydrogels are essential in contemporary regenerative medicine because they can precisely control release while fine-tuning characteristics such as mechanical stiffness, degradation rate, and biochemical signaling. Tunable hydrogels are at the forefront of coordinating the intricate biological cues necessary for effective tissue repair and regeneration, from directing stem cell differentiation to encouraging angiogenesis and integrating with host tissues [30].

### 3.3. Cell Microenvironments & Bioprinting

Regenerative medicine seeks to replicate the body’s natural repair processes, aiming to replace or heal damaged tissues. The ability to accurately replicate the cell microenvironment—that complex network of chemical and physical cues that naturally directs cell behavior, differentiation, and the eventual formation of functional tissue—is a critical component in this pursuit. When paired with advanced additive manufacturing methods, such as bioprinting, tunable hydrogels have emerged as essential materials for designing intricate microenvironments [105]. Effective regenerative therapies depend on the ability to replicate this complexity both in vitro and in vivo. Because their properties can be precisely tailored to match the various properties of various tissues—from the delicate softness of neural tissue to the rigidity of bone—tunable hydrogels perform exceptionally well in this situation [106]. Due to its natural tunability, scientists can regulate key elements such as mechanical stiffness, porosity, and degradation rates, ensuring that the hydrogel scaffold decomposes in a manner that promotes the formation of new tissue [107].

Furthermore, these hydrogels can be functionalized with particular biochemical cues, such as peptides, growth factors, and cell adhesion motifs, to actively guide cell migration, proliferation, and differentiation toward particular tissue outcomes [30].

One revolutionary technique for creating these complex hydrogel-based microenvironments is bioprinting. With the use of this sophisticated additive manufacturing technique, complex, pre-defined 3D tissue constructs can be created by precisely depositing “bio-inks”—usually hydrogels loaded with living cells—layer by layer. The potential for complex tissue engineering significantly increases when tunable hydrogels are created as bio-inks [108]. This synergy allows for the following:

1. Accurate spatial management of cell positioning: By simulating the highly ordered multicellular structures found in natural tissues, bioprinting enables the precise placement of various cell types within a scaffold. To replicate intricate organ architecture, this is essential [109];
2. Creation of complex architectures: bioprinting enables the design and construction of intricate geometries, such as internal vascular channels, as well as the production of various material distributions that replicate the subtle structural differences found in organs [110];
3. Localized microenvironmental control: It is possible to design sections of a single bio-printed construct with different hydrogel characteristics, such as different growth factor presentations or stiffness gradients. This allows for localized regulation of cell behavior, mirroring the minute changes in the surrounding environment that cells experience in a natural tissue [111].

Take the regeneration of bone as an example; to achieve this, a bio-printed hydrogel that can be adjusted in stiffness and loaded with osteo-inductive growth factors could be created. For nerve repair, on the other hand, a softer hydrogel that contains neurotrophic factors could be bio-printed into a particular conduit structure to direct the growth of axons [112]. The development of truly functional and therapeutic tissue constructs is moving closer to the ultimate goal of regenerating damaged body parts thanks to the combined power of tunable hydrogels and bioprinting [113]. However, challenges remain in striking the ideal balance between printability, mechanical integrity, and optimal biological functionality for all tissue types.

## 4. Tunable Hydrogels in Cancer Intervention

### 4.1. Precision Drug Delivery

Given the complexity and heterogeneity of tumors, precision medicine in cancer therapy seeks to deliver treatments directly to tumor sites while causing the least amount of damage to healthy tissues. In this battle, tunable hydrogels are becoming ground-breaking instruments that provide previously unheard-of control over precise medication delivery for successful cancer treatment. These intelligent biomaterials have the potential to revolutionize the way we administer treatments by being specifically designed to react to the distinct pathological conditions present in the tumor microenvironment [114]. Conventional chemotherapy frequently lacks specificity, which results in suboptimal drug concentrations at the tumor site and serious systemic side effects [115]. By functioning as advanced drug carriers that can release their cargo in a regulated, localized, and even triggered manner, tunable hydrogels get around these restrictions. They accomplish this by being naturally stimuli-responsive, which enables their physical characteristics to change in response to various stimuli [116]. According to Liu et al. [117], Yao et al. [118], Kass and Nguyen [119], and others, biocompatible hydrogels with multinetwork and porous structures have been widely used in a wide range of biomedical applications, such as bone tissue regeneration, wound healing, antimicrobial therapy, biosensing, and cancer treatment. Researchers and doctors around the world have been particularly interested in hydrogels with biodegradability, injectability, and stimuli responsiveness (for example, light, temperature, and pH) for oncology drug delivery, especially in the field of cancer therapy [120] (Table 3).

As a novel form of drug carrier, hydrogels offer numerous exceptional benefits that could revolutionize cancer treatment approaches. Firstly, hydrogels can transport drugs effectively and with precise control. To create a cascade of therapeutic modalities and enable an integrated sequence of cancer treatment, hydrogels can be loaded with a range of therapeutic agents, including immunosuppressants, radionuclides, and chemotherapeutic drugs [121].

Second, there are numerous delivery methods (intravenous injection, in situ injection, in situ implantation, transdermal delivery, oral delivery, pulmonary delivery, and trans-arterial chemoembolization) and a broad range of sizes (including macro-gels, microgels (0.5–10 μm), and nanogels (<200 nm)) for hydrogels. Therefore, the hydrogel material minimizes the quantity of medicine needed and systemic toxicity by providing continuous and regulated drug administration, enabling precise access to the cancer site [119]. Lastly, the hydrogel system can react intelligently to environmental cues both within and outside the body, allowing the anti-cancer active ingredients to be remotely controlled and released as needed. Tumors and healthy tissues have quite diverse microenvironments in terms of temperature, redox potential, reactive oxygen content, pH, and many other factors. Based on these variations, responsive hydrogels may intelligently detect tumor tissue and release medications as needed, significantly increasing the therapeutic efficacy of the loaded medications while minimizing harm to healthy tissues [122].

Chemotherapy frequently damages the human body while failing to eradicate cancer cells [123]. Hydrogels are an excellent novel drug carrier that may effectively participate in cancer medication delivery, manage drug loading and release, and have long-lasting effects. When anti-cancer medications are exposed to organic solvents, the likelihood of denaturation and aggregation is significantly decreased since hydrogels are typically created by crosslinking polymerization in aqueous solution. Hydrogels’ superior biocompatibility and biodegradability help mitigate the adverse effects of chemotherapy. When loading active medications, the superior physicochemical qualities of hydrogels can either stabilize their in vivo action or increase their tolerance in vivo [124].

A self-assembled hydrogel that responds to temperature and pH was recently created by Lee et al. [125] by combining the necessary quantity of an anti-cancer medication with a salt solvent consisting of transferrin and dithiothreitol. The temperature and pH of the solvent can be adjusted to control the release of anti-cancer drugs in hydrogels efficiently. After 48 h of incubation, 80% of cancer cells can be killed by the hydrogel, demonstrating a highly efficient inhibitory effect on cancer due to the efficient, controlled, and sustained release of anti-cancer medicines.

Hydrogels with multinetwork and polyporous architectures, on the other hand, may carry precise doses of radionuclides that can be uniformly dispersed throughout the cancer cells and frequently exhibit shape adaptability and reactivity based on a range of internal and external stimuli [126]. Furthermore, the hydrogel can efficiently transport heat-sensitive substances, photosensitizers, radiosensitizers, and chemotherapy medications simultaneously. Accordingly, the hydrogel systems can not only prevent damaged DNA from randomly repairing itself and from replicating, but they can also effectively reduce the radiation resistance state of the cancer under hypoxia, which causes multiple DNA function damages and improves the therapeutic effect of brachytherapy [127,128].

Wang et al. [129] developed an injectable hydrogel composed of hyaluronic acid-tyramine and endostatin (ES). By efficiently and reliably releasing ES, the hydrogel effectively lowers the hypoxia of cancer microvascular density and dramatically increases the cancer’s sensitivity to radiation. Zhang et al. [130] described a multifunctional hydrogel that utilizes radiation nuclide iodine-131 (131I)-labeled methoxy polyethylene glycol (mPEG), doxorubicin, and gold nanoparticle aggregates (GNPs) to enhance the effectiveness of radiation treatment.

**Table 3:** Hydrogel Approaches for Precise and Effective Drug Administration.

Method	Precision	Effects	References
**Tunable hydrogels as drug carriers**	Designed to deliver medication specifically to tumor sites based on local pathological conditions	Enhances treatment effectiveness while minimizing harm to healthy tissue	[119,120]
**Stimuli-responsive hydrogels (e.g., light, pH, temperature)**	React to specific tumor microenvironment signals to trigger drug release	Allows controlled and targeted release of therapeutic agents; improves drug efficiency	[123,127]
**Biodegradable and injectable hydrogel systems**	Enable localized administration with built-in degradation over time	Improve patient comfort, reduce systemic exposure, and simplify administration	[127]
**Multinetwork and porous hydrogel structures**	Provide channels for uniform drug distribution and adjustable loading capacity	Enhance versatility for various treatments, including wound healing, biosensing, and cancer therapy	[122,123,124]
**Hydrogels for combination therapy (e.g., loaded with chemo- and immunodrugs)**	Can carry multiple types of drugs for sequential or simultaneous release	Enable integrated cancer treatment strategies with better coordination of therapy modalities	[129]
**Hydrogels replacing conventional chemotherapy methods**	Offer localized, sustained, and responsive release versus passive, non-targeted chemotherapy	Avoid systemic side effects and ensure higher drug concentration at the tumor site	[119,120,121]

### 4.2. Immunotherapy Enhancement

Cell therapy, a cutting-edge treatment method, utilizes living cells to combat diseases and promote healing within the body. This novel method involves administering live cells for medicinal purposes by injection, transplanting, or implantation. Cell-based therapy offers inherent therapeutic benefits that surpass those of conventional medication therapies. When these biologically active cells enter the body, they can quickly adjust to and react dynamically to a variety of biological signals and physicochemical stimuli. They can also interact with the body’s cells to carry out their therapeutic roles [131]. These adaptable qualities provide them with an inherent advantage in the fight against incurable illnesses, allowing for the regeneration and repair of injured tissue or the regression of malignant tumors [132].

Traditional treatments like chemotherapy and radiation therapy have limited therapeutic benefits because cancer frequently has very complex microenvironments [133,134]. Immunotherapy techniques work by stimulating the body’s robust immune system, which helps prevent the spread and recurrence of many types of cancer. As a result, immunotherapy is among the most promising approaches to enhance the therapeutic outcomes of conventional cancer treatments, which have been extensively employed to treat various cancers [135,136]. The majority of immune checkpoint blockers used in clinical practice, however, are monoclonal antibodies with large molecular weights that have trouble penetrating and readily accumulating in solid tumors [137]. Additionally, most patients have low response rates to immunotherapy because to the low immunogenicity and inadequate infiltration of antigen-specific T cells in a variety of cancers. The development of hydrogels capable of delivering different hydrophilic medications locally can significantly improve the therapeutic outcome of cancer immunotherapy [138]. Numerous immune checkpoint blockers and substances that can boost cytotoxic T lymphocyte infiltration and cancer immunogenicity can be added to the hydrogel. Additionally, the hydrogel enables the prolonged release of these medications in a sequence that decreases drug resistance and the need for repeated injections while simultaneously triggering a series of immune responses for synergistic therapeutic advantages [139,140]. Furthermore, the hydrogel’s encapsulation promotes the long-term accumulation of medications and, to a certain degree, improves the penetration of immunotherapeutic medicines [141]. A hybrid peptide hydrogel comprising melittin, (RADA)32 titanium, and doxorubicin (DOX) was described by Jin et al. [142] for the treatment of melanoma through chemoimmunotherapy. The hydrogel’s regulated release quickly activates innate immune cells, including cytotoxic T cells and dendritic cells, while M2-like tumor-associated macrophages (TAMs) are particularly depleted. As a result of the hydrogel system’s effective reshaping of the immunosuppressive tumor microenvironment (TME), melanoma becomes particularly vulnerable to the immune system. Melittin, (RADA) 6 peptide, and KN93 (a particular Ca^2+^/calmodulin-dependent protein kinase II inhibitor) were the basis for the biocompatible hydrogel system that Dai et al. [143] created. Ascites from hepatocellular carcinoma and melanoma may be treated using the hydrogel system, which also strengthens the host’s innate and adaptive anti-tumor immune responses.

### 4.3. Three-Dimensional Cancer Models and Diagnostics

The ongoing development of three-dimensional (3D) tumor models that capture intricate physiological cues in vitro is one of several advancements necessary to improve the translational efficiency of anticancer agents. The sophistication of 3D models has advanced significantly. However, there are still many important considerations to be made in order to fully appreciate the benefits—and, more importantly, the drawbacks—of employing bioengineered 3D models in the drug discovery process. The development of cost-effective, biologically relevant systems; the meaningful compatibility of complex models and associated analysis procedures with rapid throughput screening; and the definition and validation of practical treatment response metrics are the main obstacles to using 3D models to inform important go/no-go decisions in drug development [144].

A variety of stimuli, including variations in pH, temperature, magnetic fields, and near-infrared radiation, can trigger intelligent, controlled drug release mechanisms due to recent advancements in stimuli-responsive components. The use of diagnostic imaging agents, such as radiolabeled isotopes, fluorescent dyes, and magnetic nanoparticles, significantly enhances real-time therapeutic monitoring and tumor visualization. When compared to traditional techniques, multifunctional hydrogels exhibit better therapeutic results by integrating chemotherapy, photothermal therapy, photodynamic therapy, immunotherapy, and their synergistic combinations [62].

**Figure 4:**
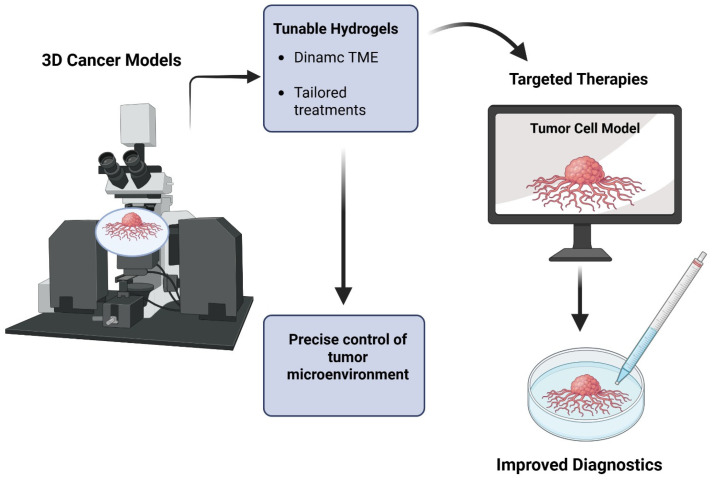
Bio-printed 3D cancer model for research and diagnosis. This figure illustrates the use of a bioprinter to create an in vitro 3D cancer model. Different cell types, such as stromal cells (grey), cancer cells (purple), and endothelial cells (blue), are precisely deposited into a structured environment using a bioprinter. By simulating physiological conditions, an integrated inflow and outflow system enables the controlled delivery of nutrients and the removal of waste products. The complex cellular architecture of the 3D cancer model is displayed in the magnified view on the right, emphasizing the interactions between stromal cells, cancer cells, and the endothelial cell-formed vasculature.

Hydrogels are three-dimensional networks of polymers that can store a significant amount of water. Due to their unique qualities and adaptability, they have become essential tools in cancer treatment. From therapeutic administration to complex 3D models and enhanced diagnostics, their intrinsic biocompatibility, adjustable mechanical characteristics, and capacity to encapsulate cells and biomolecules make them perfect platforms for a variety of cancer-related applications [145] (Figure 4).

The “tunable” component is essential, as it enables scientists to accurately replicate the dynamic and diverse tumor microenvironment (TME) and tailor treatment plans for maximum effectiveness [146].

However, due to the intrinsic non-uniformity of gel characteristics and cell population heterogeneity, which often result in varied cell responses, studying biological responses within 3D hydrogels presents significant challenges [147]. This examination is made more difficult by the introduction of degradable gel settings since it is challenging to monitor changes in gel properties in real time. Furthermore, due to difficulties in managing strain distribution inside hydrogels, existing research instruments—in particular, mechanical stretching devices—frequently fail to replicate the variety of mechanical stimuli cells faithfully experience in vivo [148]. This restriction makes it more challenging to investigate how various mechanical conditions affect cell behavior thoroughly [148]. For 3D cell research, programmable mechanical stretching devices must be designed. Microfluidic devices could be one way to accomplish this. To study the production of 3D gels and cell mechanics, microfluidics can be utilized to develop microreactors or cell culture devices that enable the mechanical manipulation of 3D cell constructs. Furthermore, microrheology may be used in the future to examine local gel breakdown processes thanks to improvements in measurement precision and active tracking technologies [147,149].

A revolutionary development in cancer research is the combination of 3D cancer models and tunable hydrogels. The development of highly effective, targeted therapies and the improvement of cancer diagnostics are being aided by these advanced platforms, which provide precise control over the tumor microenvironment.

## 5. Comparative Analysis Between Hydrogels

Materials such as natural polymers (like chitosan and alginate) and synthetic polymers (like PEG and PNIPAM) are critically evaluated in this section, with their distinct benefits and drawbacks for particular uses, like tissue engineering and drug delivery, discussed. A new table that compiles these comparisons is provided.

Natural polymers that come from biological sources include chitosan and alginate. Excellent biocompatibility, biodegradability, and a structure that frequently resembles the natural extracellular matrix are their main advantages. They are therefore very well adapted for tissue engineering, where they can offer a scaffolding that supports the development of cells and the regeneration of tissue [150]. For instance, chitosan’s hemostatic and antimicrobial qualities make it a preferred option for wound healing. Their main disadvantages, however, are frequently a lack of exact control over their mechanical characteristics and rates of degradation, which can be quite inconsistent and challenging to standardize [151].

Synthetic polymers are produced in a laboratory, including poly (ethylene glycol) (PEG) and poly (N-isopropylacrylamide) (PNIPAM). Their great degree of control over their chemical and physical characteristics is their primary advantage. Their mechanical strength, rate of degradation, and sensitivity to particular stimuli (such as pH and temperature) can all be accurately adjusted by researchers [152]. They are therefore perfect for medication delivery systems when a regulated and predictable release profile is essential. Hydrogels with adjustable degradation and low immunogenicity are commonly made using PEG, for example [153]. The main drawback of synthetic polymers is that they frequently do not have the same biological cues as natural polymers, which can be a drawback for some tissue engineering applications [154].

### 5.1. Quantitative Discussion of Trade-Offs and Limitations

A hydrogel’s mechanical characteristics (such as stiffness and elasticity) and rate of degradation must be carefully balanced for its intended biological use. A critical examination of these trade-offs goes beyond a straightforward explanation to include a mathematical analysis of how these parameters are adjusted to guarantee efficacy and safety. The application alone determines the ideal balance.

In applications such as cartilage regeneration, the hydrogel needs to give the developing tissue sustained mechanical support. The hydrogel scaffold needs to have a high compressive modulus (for example, between 100 kPa and 1 MPa) to withstand mechanical stress because cartilage is a load-bearing tissue [155]. In order to guarantee a lengthy degradation period (weeks to months) that corresponds with the sluggish rate of cartilage regeneration, these hydrogels are made with a high crosslinking density [27].

However, a hydrogel used for short-term drug delivery, for example, a localized cancer treatment, needs a distinct set of characteristics. In this case, the main objective is to release the pharmacological payload over a predetermined, frequently shorter period [28]. High stiffness can impede medication diffusion and is also unnecessary. In order to allow for faster degradation (hours to days) and regulated drug release prior to clearance from the body, these hydrogels are made to be softer (compressive modulus frequently less than 10 kPa) and have a reduced crosslinking density [96].

The quantitative trade-off between a hydrogel’s intended function and its stiffness and disintegration rate is evident. For long-term structural support, a stiff, slow-degrading hydrogel is best; for temporary, regulated therapeutic release, a soft, fast-degrading hydrogel is more appropriate. For hydrogel-based biomedical devices to be successful, this focused tuning is essential.

### 5.2. Critical Evaluation of Clinical Translation Challenges

The translation of hydrogel-based products from lab research to clinical use is a complex process with several obstacles. Hydrogels have enormous potential, but obstacles with long-term biocompatibility, large-scale production, and regulatory approval frequently keep promising materials from becoming commercially available.

The regulatory environment around hydrogels is complex since they can be categorized as biologics, medical devices, or drug–device combinations, each of which has a strict approval process. One of the main challenges is proving the hydrogel’s and its breakdown products’ long-term safety and effectiveness [156]. Hydrogels for cartilage regeneration, for example, need to demonstrate that the byproducts of their disintegration are non-toxic and do not result in chronic inflammation. This can require months or even years of in vivo testing. The unpredictability of degradation in vivo introduces a level of complexity that is challenging to duplicate in well-monitored laboratory environments [157].

One of the main bottlenecks in production is the transition from tiny, lab-bench batches to commercial quantities. It is challenging to guarantee batch-to-batch uniformity in important characteristics, including porosity, mechanical strength, and drug-loading efficiency. For instance, the synthesis of intricate smart hydrogels frequently depends on certain reaction conditions that are difficult to scale up, which results in high production costs and unpredictability. This difficulty is one of the leading causes of the failure of numerous creative hydrogel prototypes to reach the market [158].

Although a hydrogel may be biocompatible in the short term, it may not interact with the body predictably over the long run. Prolonged inflammation, immunological rejection, or the buildup of breakdown byproducts can all cause problems. For instance, hydrogels made for injectable drug administration need to break down entirely and be eliminated from the body without causing inflammation. This is a crucial component for long-term implants or items used to control chronic diseases [159].

### 5.3. Identification of Knowledge Gaps and Research Priorities

The field of hydrogel-based treatments has to fill a number of important knowledge gaps in order to progress and close the gap between laboratory-based prototypes and clinical solutions. A few crucial areas should be the focus of future study in order to fully utilize these materials.

The inability to precisely and non-invasively track hydrogel behavior in real time within a living creature is a significant drawback. To monitor important processes, including degradation rates, drug release kinetics, and the host’s immunological response, current techniques are frequently intrusive or lack the spatial and temporal precision required. The creation of sophisticated in vivo imaging methods, such as MRI, PET, or fluorescence imaging, that can offer accurate, quantitative information on a hydrogel’s performance from implantation to complete degradation is a top research objective. This would provide a more thorough comprehension of the interactions between hydrogels and intricate biological systems, guiding the development of safer and more efficient materials [160].

Even though stimuli-responsive hydrogels represent a significant advancement, the majority of existing formulations only react to one trigger, such as temperature or pH. Multiple, simultaneous stimuli are present in significantly more complicated biological settings. The development of genuinely multi-responsive hydrogels that can respond to several triggers is a top research objective. For instance, a hydrogel might be engineered to release a medication only in response to the detection of a specific enzyme (a cancer biomarker) and a low pH, which would both point to a tumor. This would minimize off-target effects and significantly improve the precision and specificity of drug delivery [19].

Hydrogels are still primarily designed empirically, using lengthy and laborious trial-and-error trials. The field must adopt a more data-driven and predictive methodology. Utilizing machine learning (ML) and computational modeling to forecast hydrogel performance based on structural characteristics and chemical composition is a significant area of research interest. Complex correlations between a hydrogel’s characteristics (such as its monomer type and crosslinking density) and its behavior in vivo could be modeled by ML algorithms trained on current information. This would streamline the whole development process by speeding up the identification and refinement of novel hydrogel formulations [161].

### 5.4. Challenges and Future Outlook

Although hydrogels have considerable potential as adjustable platforms for cancer treatment and regenerative medicine, several issues need to be addressed before their full clinical potential can be realized. Optimizing their mechanical properties to match the various physiological environments of various tissues precisely is a significant challenge. Although tough, self-healing, and stimuli-responsive hydrogels have advanced significantly, it remains challenging to ensure long-term mechanical stability and predictable rates of degradation in vivo, particularly for load-bearing applications [30].

Scalability and manufacturing consistency present yet another significant obstacle. It is necessary to overcome considerable engineering and logistical challenges in order to translate lab-scale hydrogel synthesis into large-scale, economical production for clinical use while maintaining strict quality control. Since these novel, frequently multifunctional biomaterials must pass stringent safety and efficacy tests in order to receive clinical approval, regulatory pathways also pose a significant obstacle. Concerns such as possible toxicity from unreacted monomers or degradation products as well as ensuring the product is immunogenic and biocompatible are crucial [162].

Looking ahead, the future of hydrogels is poised for transformative advancements. Research will focus on integrating multi-functionality, developing hydrogels that can not only provide structural support but also deliver drugs, genes, or cells in a precisely spatiotemporally controlled manner. The incorporation of innovative functionalities, such as responsiveness to specific biomarkers, pH changes, or external stimuli (e.g., light, ultrasound, etc.), will enhance the therapeutic precision of these devices. Furthermore, the convergence of hydrogel technology with artificial intelligence (AI) and machine learning will enable the rational design and rapid optimization of novel hydrogel formulations, accelerating their development and clinical translation [163].

Future developments in artificial intelligence (AI) and machine learning (ML) are closely linked to the potential of hydrogels as adjustable platforms for cancer intervention and regenerative medicine. These computational tools are providing answers to important problems in personalized medicine by changing the paradigm from conventional, trial-and-error experimentation to data-driven, predictive design [164].

The capacity of AI to accurately forecast the properties of hydrogels is one of its most significant uses. Numerous tests are typically needed to design a hydrogel with particular properties, such as stiffness, degradation rate, or drug release profile. To develop predictive algorithms, researchers can use machine learning models to examine large datasets of current hydrogel compositions and their corresponding attributes. After that, these models may be used to quickly test novel formulations and determine the best mix of materials, crosslinking agents, and manufacturing conditions to obtain the desired result. In addition to speeding up the R&D process, this also drastically lowers expenses and material waste [165].

AI is a key component that makes personalized medicine possible. AI models can create hydrogels tailored to a person’s needs by combining patient-specific data with databases of hydrogel properties, such as genetic markers, disease stage, and physiological characteristics. For instance, in the treatment of cancer, an AI algorithm might create a hydrogel that would release a chemotherapy medication at a pace that targets explicitly the drug metabolism and tumor growth rate of a patient. Similarly, a hydrogel scaffold for regenerative medicine might be designed to precisely match the mechanical properties of the wounded tissue in a specific patient, allowing for a quicker recovery [166].

AI and ML algorithms that predict how different stimuli would affect medication release kinetics can be used to optimize the design of these responsive systems. This level of accuracy is necessary to reduce adverse effects and increase therapeutic effectiveness. For example, to ensure a tailored and effective reaction in an infected wound, a smart hydrogel may be engineered to release an antimicrobial chemical only when it recognizes the presence of particular bacterial toxins [167].

Hydrogels’ intrinsic tunability makes them incredibly well suited for customized treatments in cancer intervention and regenerative medicine. Hydrogels are at the forefront of the movement to replace the “one-size-fits-all” approach to treatment with strategies that account for individual patient variability.

Hydrogels can be tailored to a patient’s unique tissue defects in regenerative medicine. The regeneration of specific tissues, like cartilage, bone, or skin, can be facilitated by encapsulating patient-derived cells in hydrogels with precisely regulated stiffness, porosity, and biochemical cues. This is made possible through sophisticated fabrication techniques, such as 3D bioprinting. This patient-specific design reduces rejection and maximizes functional recovery, potentially aided by imaging data to create anatomically precise scaffolds [168].

Hydrogels provide a potent way to customize drug delivery and alter the tumor microenvironment for cancer intervention. Traditional systemic chemotherapy frequently results in severe side effects and limited efficacy due to the heterogeneity of tumors among patients and even within a single tumor. To minimize systemic toxicity and maximize therapeutic concentration, tunable hydrogels can be designed to release immunomodulatory agents, gene therapies, or anticancer medications directly at the tumor site. Based on the patient’s genetic composition, drug sensitivity profiles, and tumor characteristics, this local, prolonged, and frequently stimuli-responsive release can be customized [169].

One major problem is that there are no standardized preclinical models. Simplified in vitro or animal models that fall short of accurately simulating the complexity of the human body are used in many lab experiments. Developing more advanced, multi-organ-on-a-chip platforms that can more accurately forecast a hydrogel’s function, stability, and possible toxicity in people is necessary to address the issue [170].

Additionally, the regulatory pathway for these novel, multi-component therapies, like a hydrogel that contains both medicines and cells, is frequently unclear. For the FDA and EMA to create more efficient procedures and more explicit rules, material scientists, clinicians, and regulatory agencies must work together more. For treatments that satisfy particular safety and effectiveness requirements, this can include developing special fast-track paths [171].

In conclusion, there are numerous significant obstacles to overcome when transferring hydrogels from the lab to the clinic. First, there are regulatory and biological obstacles, such as making sure the hydrogels are stable and biocompatible inside the body, which may cause immunological reactions. Another major obstacle is the costly and time-consuming regulatory approval procedure, particularly for complicated materials. Secondly, it is challenging to scale up manufacturing and quality control. To provide predictable treatment outcomes, it is crucial to maintain consistent characteristics throughout many production batches, and strong quality control procedures are required. Developing more precise in vitro models to minimize animal testing and producing customized hydrogels for each patient should be the main goals of future research. Hydrogels can realize their full potential in clinical practice if smart hydrogels that react to biological stimuli are developed [172].

Finally, it is important to consider early in the research phase if large-scale production is economically feasible. A hydrogel’s economical and scalable manufacture should be a priority for researchers beyond its biological role. The development of automated, closed-system manufacturing techniques may guarantee uniformity, lower production costs, and minimize contamination concerns, hence expanding the patient base for these treatments [173].

## 6. Conclusions

Hydrogels, a new class of biomaterials, provide unparalleled accuracy and versatility in advanced medical applications, especially in precision medicine and regenerative engineering. Their water-rich, porous, and highly biocompatible nature essentially mimics the body’s natural ECM, making them perfect for interacting with biological systems in a seamless way without causing negative immune reactions.

The power of hydrogels lies in their tunable nature. By carefully selecting the appropriate materials and employing various crosslinking techniques (both physical and chemical), their mechanical properties, rates of degradation, and chemical functions can all be precisely designed. This makes it possible to customize stiffness to fit particular tissues (like cartilage versus brain), control swelling behavior for optimal medication release, and incorporate bioactive compounds to direct cellular responses.

These days, hydrogels can act as “smart” systems that react to particular internal or external stimuli, allowing for localized and on-demand therapeutic delivery. Because of this sophisticated control, hydrogels are no longer considered passive materials. This approach lessens the serious systemic toxicities associated with conventional therapies while also greatly increasing therapeutic efficacy. Moreover, their ability to model tumor microenvironments in vitro provides crucial resources for understanding the progression of cancer and developing innovative treatments.

Hydrogels provide a significant advancement in cancer treatment by enabling the prolonged and targeted delivery of anti-cancer drugs to tumor sites. Hydrogels are highly adaptable scaffolds and delivery systems for regenerative medicine that have been scientifically validated. They can be made to resemble the intricate biological environment in order to guide stem cell differentiation, promote tissue growth, and facilitate healing. Their ability to precisely control molecular signaling, cell delivery, mechanical properties, degradation kinetics, and architectural design using advanced techniques such as 3D bioprinting is essential. Current limitations could be overcome and even more potential unlocked with further advancements, especially in the production of granular hydrogels and the replication of the complex natural extracellular matrix.

Ultimately, the development of intelligent and bio-functional hydrogels represents a significant advancement. These materials give previously unheard-of control over tissue engineering, drug delivery, and cell encapsulation by fusing precise biological signals with dynamic reactivity to stimuli. Hydrogels’ synergistic combination of biomimicry and intelligent functioning makes them essential materials with enormous potential to influence medicine in the future and enable genuinely customized and efficient treatments.

## 7. Materials and Methods

This review provides a comprehensive and critical assessment of the current state of tunable hydrogels in cancer treatment and regenerative medicine. With a focus on key aspects of hydrogel design, properties, and applications, the methodology employed in this study involved a comprehensive and methodical review of the existing scientific literature.

*Search for Literature and Selection Method*

A wide range of peer-reviewed scientific articles, reviews, and pertinent publications were used to compile the data in this review. Studies that were given priority during the selection process were those that discussed the synthesis and basic characteristics of hydrogels and discussed the field’s obstacles, potential, and prospects.

The following were some of the key terms and their combinations that were used in the literature search: “hydrogels”, “tunable hydrogels”, “smart hydrogels”, “bio-functional hydrogels”, “precision medicine”, “regenerative medicine”, “tissue engineering”, “controlled drug release”, “cell microenvironments”, “3D bioprinting”, “cancer therapy”, “tumor microenvironment”, “crosslinking”, “mechanical properties”, “swelling behavior”, “degradation kinetics”, and “porosity”.

*Types of Hydrogel Materials*

The review summarizes data from several classes of hydrogel materials, including both synthetic and naturally occurring polymers. Frequently discussed resources include the following:

Natural polymers include hyaluronic acid (HA), collagen, chitosan, peptides, alginate, fibrin, gelatin, elastin, and silk protein.

Synthetic polymers include hydrogels based on polyacrylamide, polyvinyl alcohol (PVA), and polyethylene glycol (PEG).

*Methods for Hydrogel Fabrication and Tuning*

The review describes the different methods used to prepare and precisely adjust the properties of hydrogels, which are essential for their wide range of biomedical applications.

*Techniques for Crosslinking*

Chemical crosslinking includes enzymatic crosslinking (e.g., citric acid for CMC/PVA), click chemistry, photo-crosslinking, and free radical polymerization. Physical crosslinking includes van der Waals forces, hydrogen bonds, ionic interactions, hydrophobic associations, and physical entanglement (e.g., methylcellulose-based, alginate-based, etc.).

*Methods for Figures*

Figures were created using Bio Render (https://BioRender.com/8fd1f6o (accessed on 09, 10 August 2025)).

## Figures and Tables

**Table 1:** Hydrogel Properties and Control Mechanisms.

Hydrogel Property	Physico-Chemical Control	Mechanical Control	References
**Mechanical Strength**	Monomer composition, molecular weight, crosslinking chemistry, incorporation of hydrophobic/ionic domains	Crosslinking density, thermal annealing, nonsolvent quenching, hierarchical structuring	[27,28,43]
**Elasticity and Viscoelasticity**	Polymer network structure, entropic elasticity, fiber alignment	Degree of crosslinking, hierarchical fibrous networks, morphology type (rubber-like vs. fibrillar)	[26,28,36,37,43]
**Swelling Behavior**	Hydrophilic/hydrophobic balance, ionic interactions, degree of ionization	Crosslinking ratio, network stiffness, pore size	[23,26,48]
**Degradation Rate**	Degradable linkers (e.g., ester, enzyme-sensitive), pH-responsive groups, self-cleaving linkers	Crosslinking density, porosity, network structure	[22,29]
**Porosity**	Fabrication technique (e.g., solvent casting, gas foaming, freeze drying), microfabrication for channel creation	Scaffold stiffness (higher porosity generally leads to lower stiffness)	[36,37]
**Drug Release Kinetics**	Polymer–drug interactions, ionic content, degradation mechanism, nanoparticle/microsphere incorporation for staged release	Swelling capacity, porosity, crosslinking level, compartmentalized structures	[28,31,47,57]
**Microarchitecture**	Microfabrication (e.g., soft lithography, prototyping), material chemistry, hierarchical multi-scale designs	Pore design, structural alignment, integration of micro/nanoparticles	[33,34]
**Integrated Design Trade-offs**	Polymer chemistry to balance hydration, degradation, and diffusion	Trade-offs between stiffness, porosity, swelling, and support	[27,28,36]

**Table 2:** Hydrogel Properties and Their Contributions to Regenerative Medicine.

Hydrogel Property	Benefit	Limitation of Conventional Materials	References
**High water content**	Mimics the natural extracellular matrix (ECM) and supports cell viability	Hydrophobic or have a low water content	[70]
**Biocompatibility**	Promotes safe integration with host tissues without immune rejection	Immunological responses, inflammation, or foreign body reactions	[70,77]
**Tunable chemical and physical properties**	Allows precise control of cell behavior (migration, proliferation, differentiation)	Fixed and challenging to modify	[71,72,73]
**Spatiotemporal control of biophysical cues**	Enables dynamic control of stiffness, porosity, and degradation to guide tissue formation	Unable to replicate the biological environment’s dynamic signals	[72,73]
**Incorporation of functional ingredients**	Enhances biochemical signaling for improved tissue regeneration	Embedding fragile biological molecules is challenging	[74]
**Nano- and micropatterning capabilities**	Facilitates complex multicellular designs and studies of cell–matrix interactions	Needs costly, multi-step lithography	[75]
**Customizable architecture and mechanics**	Supports development of ECM-mimetic structures tailored to specific tissue types	Conventional materials’ final mechanical stiffness and form are mostly determined during manufacture	[76]
**Collagen-based formulations**	Naturally cell-binding and supportive for tissue regeneration	Removing the natural biological cues	[77,78]
**Composite and crosslinked systems**	Improve stability, mechanical strength, and durability of hydrogel scaffolds	Non-biodegradable or brittle materials	[79,80,81,82]
**Granular hydrogels (GHs)**	Provide high porosity, injectability, and nutrient/waste exchange	Solid and monolithic rather than composed of linked microparticles	[86,87,88]
**Customizable hydrogel microparticles (HMPs)**	Allow fine-tuning of mechanical properties and improved cell migration	Stop cell integration, migration, and infiltration	[86,87,88]
**Advanced fabrication methods (e.g., bioprinting)**	Overcome limitations of traditional hydrogels and support structural complexity	Incompatible with the sensitive parts	[91,92,93,94]
**Regulated degradation rates**	Synchronizes scaffold breakdown with tissue regeneration pace	Non-biodegradable or breaking down uncontrollably	[49,50,51]
